# Properties Evaluation of Different Graphite Brands through the Development of Electrochemical Sensors for Phenolic Compounds Detection

**DOI:** 10.1021/acsomega.4c10281

**Published:** 2025-02-04

**Authors:** Amanda Neumann, Luiz Otávio Orzari, Juliano Alves Bonacin, Bruno Campos Janegitz

**Affiliations:** † Laboratory of Sensors, Nanomedicine, and Nanostructured Materials, Federal University of São Carlos, Araras, São Paulo 13600-970, Brazil; ‡ Department of Physics, Chemistry and Mathematics, Federal University of São Carlos, Sorocaba, São Paulo 18052-780, Brazil; § Institute of Chemistry, University of Campinas, Campinas, Sao Paulo 13083-859, Brazil

## Abstract

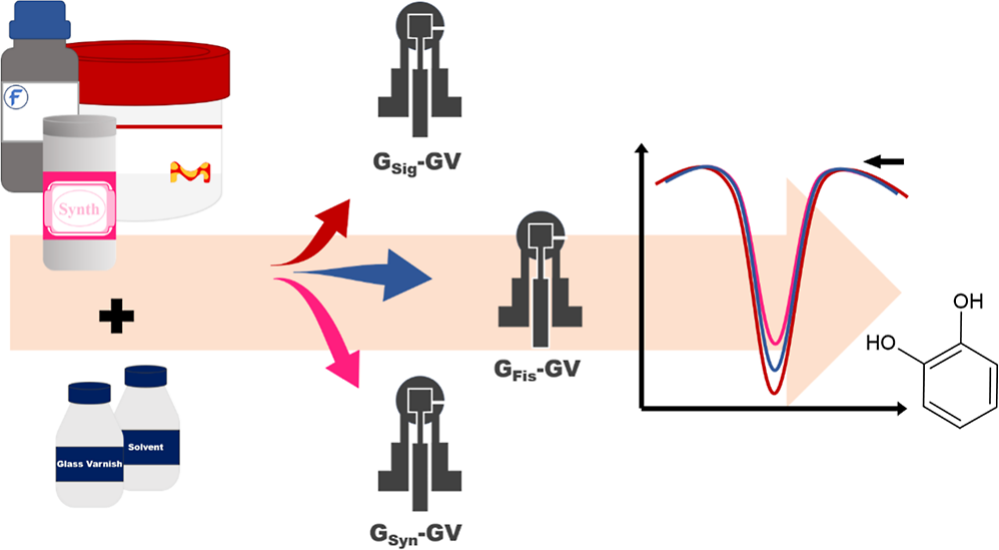

Graphite is one of the most utilized materials for producing conductive inks for screen-printed electrodes (SPEs). Because of their wide range of applications, several brands are available in the market. Consequently, it is possible to find particles with different properties. In this context, these differences can generate variations in the device performance. Based on this, the present work compares paper analytical devices (PADs), which were prepared with three different graphite brands and photographic paper, to evaluate how these possible distinctions can affect the devices’ performance. CA detection was carried out with square wave voltammetry, where the devices produced with Fisher Chemical and Sigma-Aldrich graphite stood out for their high stability and current magnitude. Under the optimized parameters, analytical curves were constructed for each PAD, resulting in 5.0 × 10^–6^ to 1.0 × 10^–4^ mol L^–1^ and 5.0 × 10^–7^ to 1.0 × 10^–4^ mol L^–1^ for that manufactured with Fisher Chemical and Sigma-Aldrich graphite, respectively. In addition, the limits of detection were 1.39 μmol L^–1^ for the one produced with Fisher Chemical graphite and 0.288 μmol L^–1^ for the one produced with Sigma-Aldrich graphite.

## 1 Introduction

Graphite is a widely studied material due to its conductive properties, relatively low cost, and chemical inertness. It has ample applications, standing out in the composition of conductive inks for electrochemical sensors.^1−3^ Its intrinsic properties may change according to the natural source, processing methods, or synthetic routes.^4−7^ Consequently, these variations can directly interfere with their electrochemical behavior, where even small differences can cause irregularities in the surface area or electrical conductivity. Additionally, other factors such as the presence of defects, impurities, and different functional groups can also influence their behavior, potentially affecting the wettability of the material as well.^8−10^

This material has a three-dimensional structure and is composed of graphene lamellas overlapping in parallel, which allow the existence of delocalized π electrons over the structure, responsible for the conductive characteristic.^11,12^ Due to its disposition, it is considered an anisotropic material, which has greater electrical conduction power when analyzed along the lamellae.^13,14^ It is important to highlight that alternative ways to obtain graphite can lead to differences in its characteristics, such as granulometry and conductivity. Therefore, distinct products can present, even minimal, particular particle size, crystallinity, and/or arrangement of these layers.^15,16^

Graphite is widely applied in sensor production, particularly in the formulation of conductive inks. These inks are mainly composed of a conductive material, a polymer, and, if necessary, additives.^2,17,18^ A notable example is screen-printed electrodes (SPEs), which use a screen to print the device in the desired geometry.^19^ In many of these SPEs, paper is employed as a substrate, commonly found in literature as electrochemical paper-based analytical devices (ePAD).^20−23^ Paper application advantages include a higher rate of degradation, vast market availability, relatively low cost, and flexibility.^3,24−27^ Thus, these devices are viewed as disposable electrochemical tools, offering portability, miniaturization, and less sample waste compared to traditional analytical equipment.^24,25,28^

The literature presents examples of the efficient use of paper as a substrate for the fabrication of electrochemical sensors, such as adhesive paper, which has proven to be a promising alternative for various applications, especially as a substrate for electrochemical systems due to its biocompatibility and biodegradability.^29^ It has been applied in the fabrication of an SPE with an efficiency for detecting serotonin and dopamine, and it has also been modified for glucose biosensing. There is also a system developed with waterproof paper, demonstrating that the hydrophobic properties of the substrate prevent samples from being absorbed by the fibers, presenting interesting characteristics for the development of such systems.^3^

In this context, to produce a sensitive, effective, and reproducible SPE it is important to evaluate different graphite brands available in the market. This study analyzes three different graphite brands for ink formulation, with photographic paper as a substrate which has a shiny layer of silicate resin, contributing to diminish the solution absorption.

Another way to evaluate graphite in ePAD involves the electrochemical validation of catechol (CA) detection and quantification, which is critical due to the need for reliable devices to monitor this harmful substance. CA is a phenolic compound derived from benzene and is highly toxic, with bioaccumulation potential and a low degradability rate.^30−32^ Due to its properties, this substance is included in the list of priority pollutants in environmental agencies in the United States of America and Europe, requiring continuous monitoring for CA.^33^

This paper presents the evaluation of Fisher Chemical, Sigma-Aldrich, and Synth graphite, selected by their availability in Brazil, for sensor performance, examining potential variations between the brands and their implications for ePAD development and CA detection.

## 2 Experimental Section

### 2.1 Reagents and Solutions

Three graphite brands analyzed in this work were purchased from Fisher Chemical, Sigma-Aldrich, and Synth. In the context of sensor production, all the materials were purchased from a local market: glass varnish and alkyd resin were from Acrilex; the substrate used was photographic paper from Scitop; and the nail polish for delimitation of the sensor working area was from Cora. Other reagents, including those for the supporting electrolytes, electrochemical probe, and CA were obtained from Sigma-Aldrich and/or Synth, in analytical grade. All the solutions were prepared with Milli-Q ultrapure water (resistivity >18.2 MΩ cm^–1^, obtained by a purification system Millipore, Synergy).

### 2.2 Apparatus

Ink homogenization was performed by a double asymmetric centrifuge SpeedMixer DAC 150.1 FVZ-K (FlackTec Inc.). The design of the sensors was made in an adhesive mask with a cutter printer Silhouette Cameo 4 and Silhouette Studio software. BEL Engineering W3B was used for all pH determinations in the parameter optimization study. Electrochemical measurements were made using a potentiostat/galvanostat PGSTAT204 Metrohm (Eco Chemie) controlled by Nova 2.1.4. software, and all experiments were conducted by adding 140 μL of the specific solution to the surface area of the sensor.

Morphological characterization of the sensors was performed using scanning electron microscopy (SEM), with a Thermo Scientific Prisma E (Waltham, MA, USA) microscope (voltage acceleration of 10 kV and low mode vacuum at 50 Pa). Spectroscopic characterization was performed by Fourier transform infrared spectroscopy (FT-IR) with a Tensor II spectrophotometer (Bruker) in 4.0 cm^–1^ resolution and transmittance mode from 4000 to 520 cm^–1^. The samples were prepared in a KBr tablet with the mass ratio of 1:100. X-ray diffraction (XRD) measurement was done with a Rigaku X-ray powder diffractometer, MiniFlex 600, with a CuKα radiation source (λ = 0.15406 nm), in an angular range of 3.0–90°, with 0.02 step and 10° min^–1^ analysis time. The contact angle analyses were performed with lab-made equipment described in ref (34). Electrochemical impedance spectroscopy (EIS) and cyclic voltammetry (CV) were used in electrochemical characterizations. Square wave voltammetry (SWV) was used for CA detection, and the studied values were 5.0 to 10 for pH, −1.0 to 10 mV for step (*s*), 5.0 to 50 Hz for frequency (*f*), and 10 to 60 mV for amplitude (*a*).

### 2.3 Production of the Disposable Electrodes

**Figure 1:**
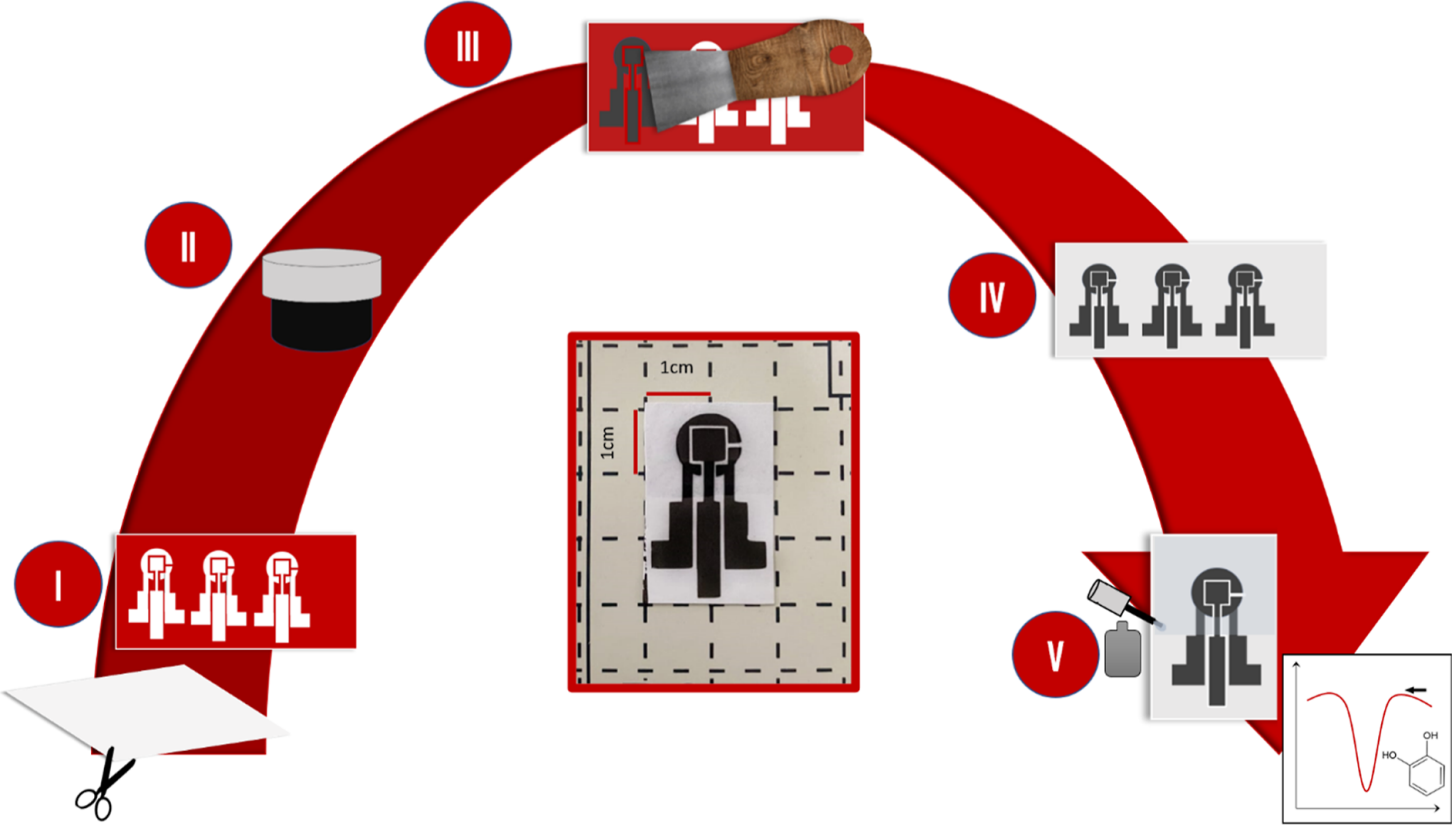
Preparation of the disposable electrochemical sensors. Cutting the substrate and attaching the adhesive mask (I). Conductive ink preparation (II) and spreading it over the adhesive mask (III). Removing the adhesive mask and the excess ink (IV). Defining the drop area with nail polish and the device is ready for the subsequent analysis (V).

The systems were fabricated by serigraphy utilizing photographic paper, as demonstrated in Figure 1. The photographic paper was cut to attach the adhesive mask (Figure 1(I)). The conductive ink was prepared with graphite, glass varnish, and an alkyd diluent, following the work of Pradela-Filho et al.^35^ (Figure 1(II)) and spread over the mask with the aid of a spatula (Figure 1(III)). After the ink was dried, the adhesive mask was removed (Figure 1(IV)), the sensors were separated, and the system was delimited with nail polish (Cora©); after the cure process, the device was ready for CA measurements Figure 1(V). In the center of the image, the real device is shown with a 1 cm scale.

This process was repeated for the three graphite brands analyzed, forming three different devices: G_Fis_-GV, G_Sig_-GV, and G_Syn_-GV, as shown in Figure S1, which were fabricated using the graphite brands Fisher Chemical, Sigma-Aldrich, and Synth, respectively.

### 2.4 Sample Preparation

The sample was prepared by adding 1.0 × 10^–2^ mol L^–1^ of CA to tap water, collected in Araras-SP (22°18′43″S 47°23′0″W). After this process, the solution was diluted with phosphate buffer (PB) at 0.2 mol L^–1^ (pH 8.0) for the spike and recovery measurements.

## 3 Results and Discussion

### 3.1 Morphological Characterizations

**Figure 2:**
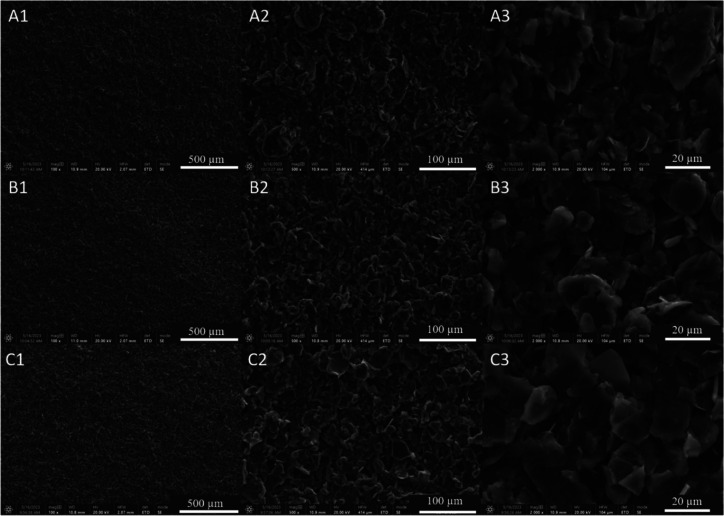
Scanning electron microscopy (SEM) images for (A) G_Fis_-GV, (B) G_Sig_-GV, and (C) G_Syn_-GV in magnifications of (1) 100, (2) 500, and (3) 2000 ×.

All three surfaces were characterized by different techniques, such as SEM, XRD, FTIR, and contact angle, aiming to analyze the structural and morphological differences generated by the different compositions of ePAD. Figure 2 presents the images obtained by SEM analyses for G_Fis_-GV [Figure 2A(1)–A(3)], G_Sig_-GV [Figure 2B(1)–B(3)], and G_Syn_-GV [Figure 2C(1)–C(3)], with different magnifications between the columns 1, 2, and 3.

In Figure 2, it is possible to conclude that all devices presented a rough surface with homogeneous carbonaceous structures. Slightly smaller clusters are in G_Sig_-GV, as can be seen in Figure 2B(2),B(3). The other two devices presented greater lamellar character, as shown in Figure 2A,C. This shows a structural difference between the surfaces, even in small proportions.

**Figure 3:**
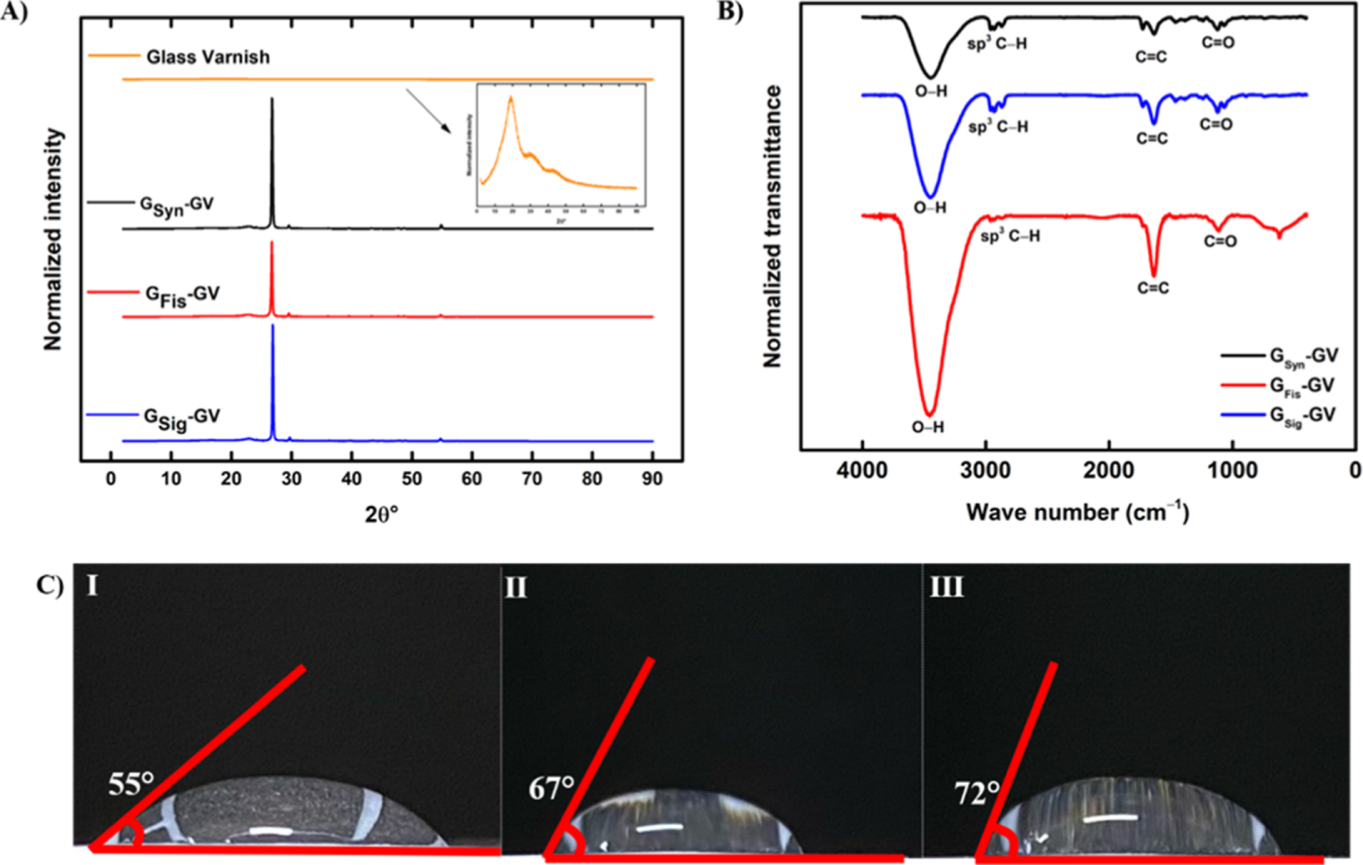
(A) XRD patterns of G_Fis_-GV (red), G_Sig_-GV (blue), G_Syn_-GV (black), and glass varnish (orange), XRD pattern of glass varnish inserted. (B) FT-IR spectra of the sensors G_Fis_-GV (red), G_Sig_-GV (blue), and G_Syn_-GV (black). (C) Contact angle images obtained by (I) G_Fis_-GV, (II) G_Sig_-GV, and (III) G_Syn_-GV.

Figure 3A presents the XRD patterns for the three ePAD and glass varnish. This characterization was performed to analyze the crystallinity of these materials due to its relation with the conductive properties. Graphite patterns in literature^36,37^ present five different plans, but in this work, only two of them are present in all the devices: (002) at 2θ = 26.5 and (004) at 2θ = 54.7°, due to the structure formed by the ink composition. Besides that, two other peaks are found in all XRD patterns at 2θ = 20 and 30°, which can be attributed to glass varnish. These amorphous diffraction patterns (insert presented in Figure 3A) are characteristic of glass-type materials and are commonly found in the literature.^38,39^

In this analysis, all three devices showed a predominant peak corresponding to (002) with a small angle variation between them. This can occur due to the difference in the crystallite size, affecting the displacement of light. These sizes were calculated by eq 1
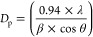
1where *D*_p_ is the average crystallite size, β is the line broadening in radians, θ is the Bragg angle, and λ is the X-ray wavelength; in ascending order: 33.79 nm for G_Syn_-GV, 41.37 nm for G_Fis_-GV, and 51.63 nm for G_Sig_-GV. For (004), there was no significant change in crystallite sizes, and probably because of that, no significant variation in the angle was observed. The difference seen in (002) suggests different interactions between the graphite and glass varnish, resulting in slightly distinct composites, as presented in the SEM analyses. In addition to these factors, G_Sig_-GV showed increased intensity in the predominant peak, which suggests greater crystallinity, followed by G_Syn_-GV and G_Fis_-GV. This variation suggests a difference in the conductivity of these devices.

These conductive inks were submitted to FTIR analysis, and the spectra acquired were similar to those obtained by Pradela-Filho et al.^35^ This was expected since they employed a similar ink formulation. In Figure 3B, it is possible to observe the presence of a broad and intense band at 3400 cm^–1^^40^, this may be associated with hydrogen bonding or polymeric O–H stretch, possibly even due to the presence of water in the analyzed materials.^41^ Two bands in these analyses refer to glass varnish according to the work of Pradela-Filho et al.,^35^ observed at 2900 cm^–1^, which can be identified as C–H bond stretching in sp^3^ carbons, and another at 1110 cm^–1^, correlated to C–O stretch.^40^ In 1630 cm^–1^, the presence of stretch C=C can be noted, referring to alkenes in the structure.^35,40,41^

Subsequently, contact angle analyses were made with the three ePAD, adding 140 μL of deionized water to each surface, to compare the hydrophobicities of these devices. The data are demonstrated in Figure 3C, where it is possible to observe the angles formed by the water–surface interaction on G_Fis_-GV, G_Sig_-GV, and G_Syn_-GV [Figure 3C(I–III), respectively]. The presented values of contact angles showed slight variations: 55° for G_Fis_-GV, 67° for G_Sig_-GV, and 72° for G_Syn_-GV. This difference is probably due to differences observed in FTIR analysis, where sensors with lower intensity bands exhibit fewer hydrophilic properties, demonstrated by the high contact angle. All devices are still considered hydrophilic according to the values established in the literature.^42^ These results are probably related to the fact that we have more or less oxygenated groups in the different graphite brands, that could be seen in XPS, but unfortunately, we do not have this equipment available.

### 3.2 Electrochemical Characterizations

**Figure 4:**
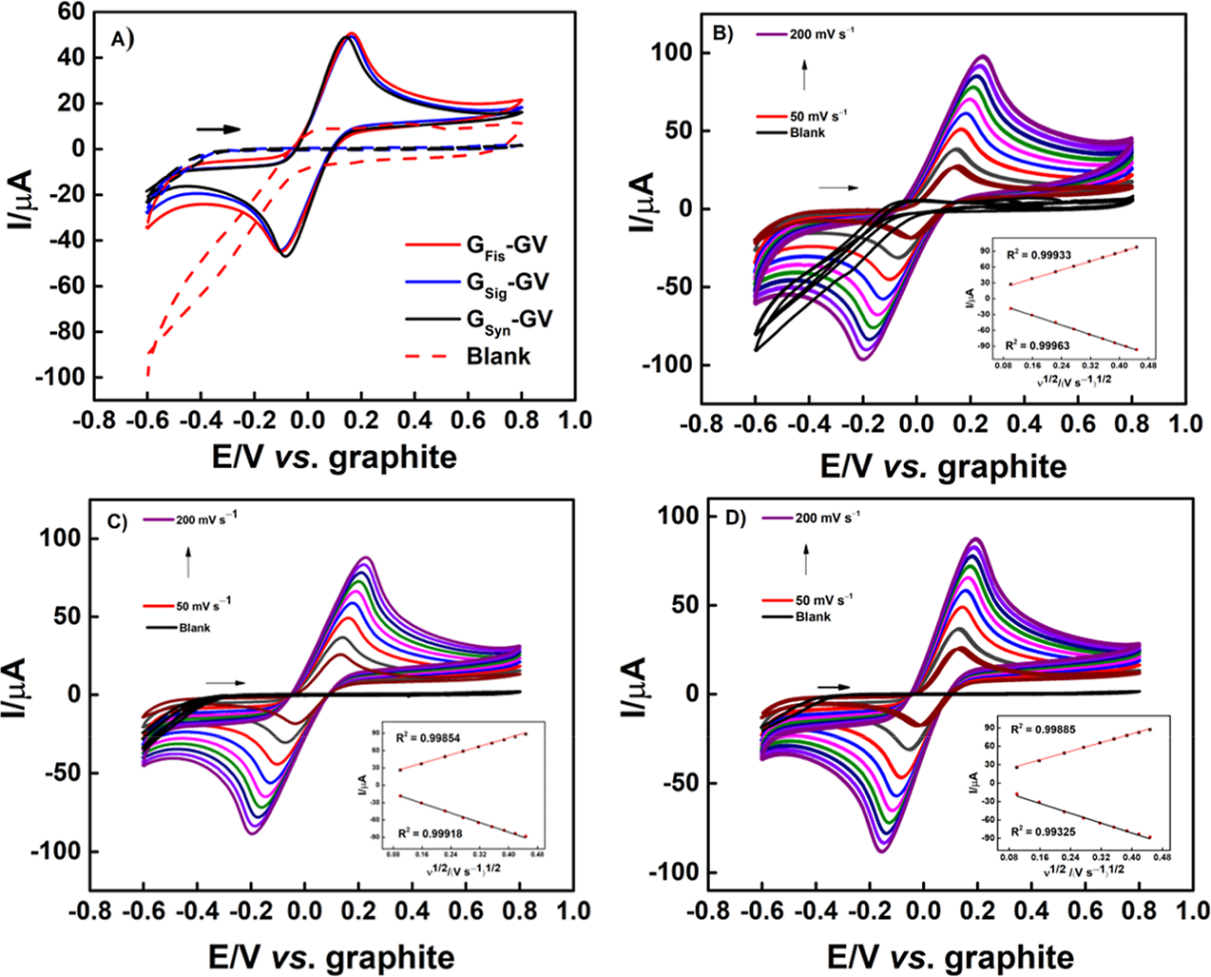
Cyclic voltammograms obtained by (A) G_Fis_-GV (red), G_Sig_-GV (blue), and G_Syn_-GV (black), in the absence and presence of 1.0 × 10^–3^ mol L^–1^ FcMeOH, in 0.1 mol L^–1^ KCl; ν = 50 mV s^–1^; (B) G_Fis_-GV in the absence and presence of 1.0 × 10^–3^ mol L^–1^ FcMeOH, in 0.1 mol L^–1^ KCl; ν = 50 to 200 mV s^–1^ and I vs ν^1/2^ correlation inserted; (C) G_Sig_-GV in the absence and presence of 1.0 × 10^–3^ mol L^–1^ FcMeOH, in 0.1 mol L^–1^ KCl; ν = 50 to 200 mV s^–1^ and *I* vs ν^1/2^ correlation inserted; (D) G_Syn_-GV in the absence and presence of 1.0 × 10^–3^ mol L^–1^ FcMeOH, in 0.1 mol L^–1^ KCl; ν = 50 to 200 mV s^–1^ and *I* vs ν^1/2^ correlation inserted.

The electrochemical behavior of the three ePAD was studied using CV and EIS. In the CV experiment, 1.0 × 10^–3^ mol L^–1^ ferrocenemethanol (FcMeOH) was used as an electrochemical probe in 0.1 mol L^–1^ KCl, with a scan rate of 50 mV s^–1^. Figure 4A shows the voltammograms for all of the devices, which do not present a significant potential peak variation. They presented anodic peaks around 156 mV and cathodic ones close to −96.3 mV, with a peak-to-peak separation (Δ*E*_p_) of 266 mV, 264 mV, and 227 mV for G_Fis_-GV, G_Sig_-GV, and G_Syn_-GV, respectively. The quotient between anodic and cathodic peak currents (*I*p_a_/*I*p_c_) for all the sensors was close to 1.0 μA, with 1.13, 1.11, and 1.04 μA, following the same order previously established. With these values of Δ*E*_p_ and *I*p_a_/*I*p_c_, it is possible to consider these redox processes as quasi-reversible electrochemical reactions.^43^ Additionally, in the G_Fis_-GV blank analysis (Figure 4A, red dashed line), a higher capacitive current is visible, when compared to the other devices. This is likely due to the difference in the arrangement of graphite in this specific composite, which influences directly in electron transfer and also for its charge-holding capacity.

In this context, experiments were carried out to evaluate the electrochemical behavior by increasing the scan rate (ν) during CV, ranging from 50 to 200 mV s^–1^, under the same experimental conditions as shown in Figure 4A. These voltammograms are presented in Figure 4B–D for G_Fis_-GV, G_Sig_-GV, and G_Syn_-GV, respectively. The current (*I*) vs ν^1/2^ correlation is shown as the inset for each system. The voltammograms show that all of these devices presented a linear increment of both current magnitude and Δ*E*_p_ with the increase of ν, indicating diffusion as the predominant mass transfer process. This is further demonstrated by the linearity of the anodic and cathodic process in G_Fis_-GV of 0.999 and 0.999, in G_Sig_-GV of 0.998 and 0.999, and in G_Syn_-GV of 0.998 and 0.993. Using these data, the electroactive areas were calculated with the aid of the Randles–Ševčík equation for quasi-reversible systems (eq 2)^44^
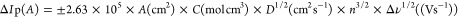
2where *I*_p_ represents the peak magnitude of current, *A* is the electroactive area to be calculated, *C* is the concentration and *D* is the diffusion coefficient of the electrochemical probe, *n* is the number of electrons, and ν is the scan rate, which showed values of 0.299 cm^2^ for G_Fis_-GV, 0.271 cm^2^ for G_Sig_-GV, and 0.260 cm^2^ for G_Syn_-GV. This area variation may be caused by a slight disparity between the size and agglomeration in each device, as shown in SEM analysis, in addition to the difference in crystallinity observed in XRD analysis.

**Figure 5:**
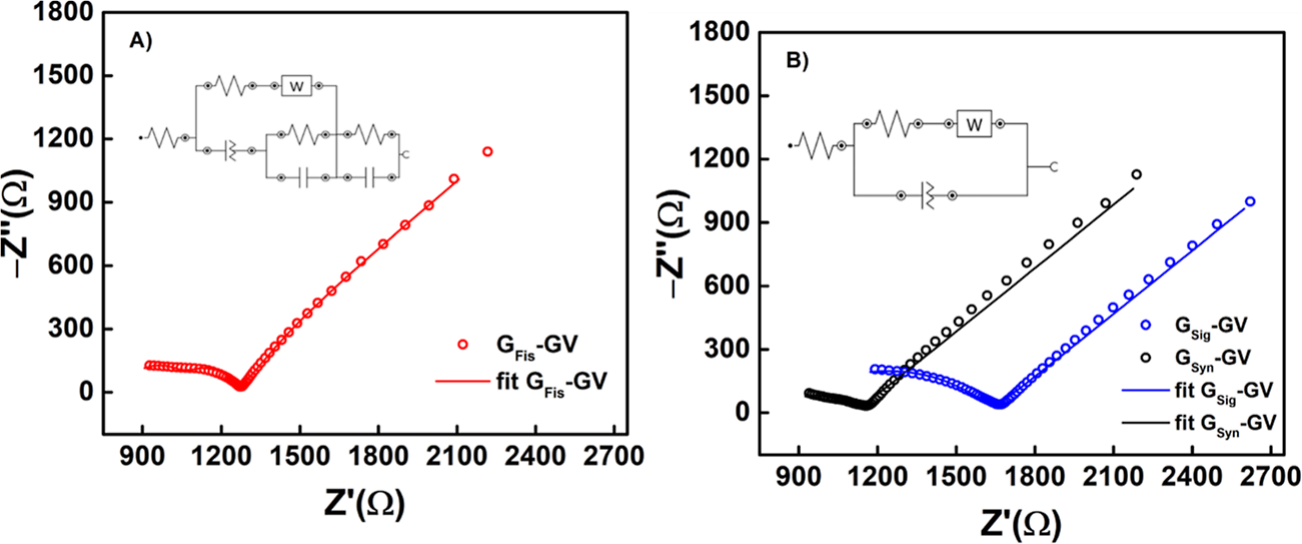
(A) Nyquist diagram obtained by G_Fis_-GV (red), in the presence of 1.0 × 10^–3^ mol L^–1^ FcMeOH, in 0.1 mol L^–1^ KCl; *E*_1/2_ = 0.088 V, equivalent circuit inserted: [*R*([RW][Q(RC)])(RC)]. (B) Nyquist diagram obtained by G_Sig_-GV (blue) and G_Syn_-GV (black), in the presence of 1.0 × 10^–3^ mol L^–1^ FcMeOH, in 0.1 mol L^–1^ KCl; *E*_1/2_ = 0.088 V, equivalent circuit inserted: [R([RW]Q)].

To analyze the electrochemical behavior of the sensor resulting from the interaction between the surface electrode and the solution, the EIS technique was used, as presented in Figure 5A, obtained by G_Fis_-GV, and in Figure 5B, obtained by G_Sig_-GV and G_Syn_-GV. The data were obtained in a frequency range of 1.0 × 10^5^ to 1.0 × 10^–1^ Hz, at 10 points per decade, 10 mV of amplitude, and sinusoidal type waves. The potential applied was 0.088 V as it is the value of the half-wave potential for all of the sensors. In all three cases, there is a capacitive interaction at higher frequencies, represented by displacement parallel to the *x*-axis. After that, the behavior is shown to be of diminished capacitance, as shown by the decrescent pattern of the points until the mass transport became controlled mainly by diffusion (Warburg impedance). These devices showed a disparity in behavior, in terms of total resistance, which is lower for G_Syn_-GV, with 217 Ω, followed by G_Fis_-GV with 304 Ω, and G_Sig_-GV with 492 Ω. Figure 5A,B insets show the equivalent circuits for the systems of G_Fis_-GV (Figure 5A) and G_Syn_-GV and G_Sig_-GV (Figure 5B). All of them consist of different modifications of a Randles circuit, to incorporate the Warburg impedance. G_Fis_-GV presented an extensive modification of the standard model possibly due to the presence of different phases. The outer pair of capacitance and resistance in the circuit of G_Fis_-GV is attributed to exposed graphite, while the internal pair suggests the presence of pores. The third pair is attributed to a different phase, probably due to the graphite and glass varnish interaction.

### 3.3 Electrochemical Detection of Catechol

**Figure 6:**
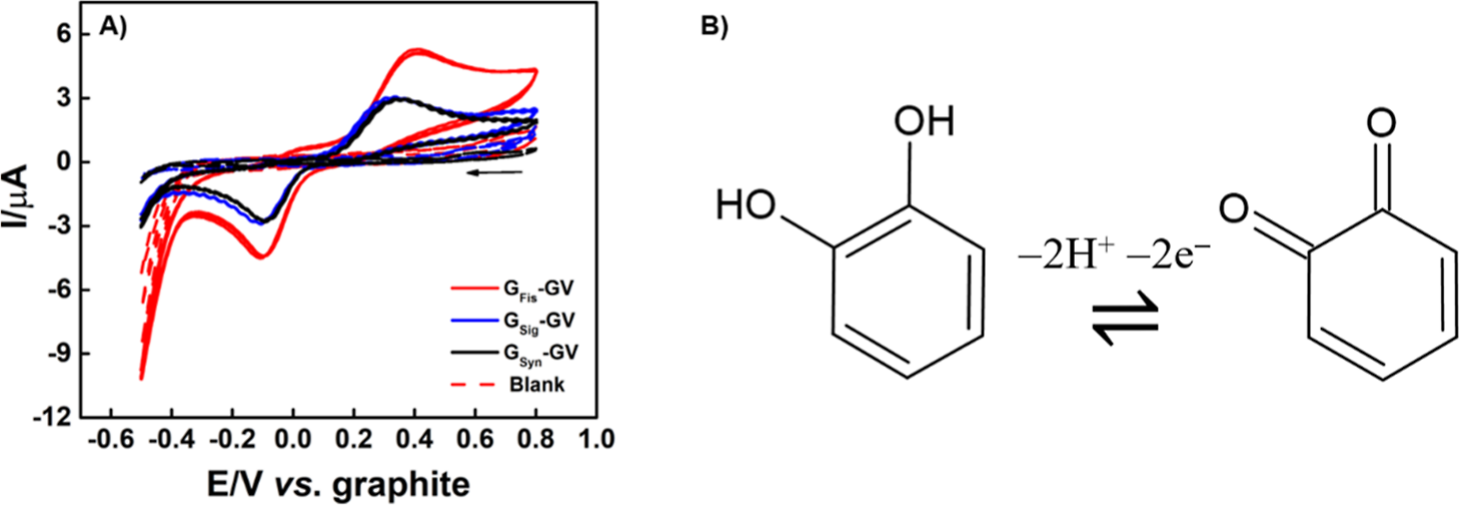
(A) Cyclic voltammograms obtained by G_Fis_-GV (red), G_Sig_-GV (blue), and G_Syn_-GV (black) in the absence and presence of 1.0 × 10^–4^ mol L^–1^ CA, in 0.2 mol L^–1^ PB (pH 7.0); ν = 50 mV s^–1^. (B) Proposal mechanism of CA redox.

The electrochemical profile of 1.0 × 10^–4^ mol L^–1^ CA in 0.2 mol L^–1^ PB (pH 7.0) was analyzed by CV at 50 mV s^–1^Figure 6A shows the results obtained in this experiment. All three sensors presented two peaks, one anodic and one cathodic, corresponding to the oxidation and reduction process, respectively. G_Fis_-GV stood out in terms of current magnitude, with peaks at 407 and −93.5 mV. Otherwise, G_Sig_-GV and G_Syn_-GV presented similar values of current magnitude and peak potentials at approximately 347 and −98.3 mV. In all sensors, the *I*p_a_/*I*p_c_ values were close to 1.0 μA, with 1.15 μA for G_Fis_-GV, 1.04 μA for G_Sig_-GV, and 1.05 μA for G_Syn_-GV. These variations of current magnitude between the devices can be justified by their different affinity with water, represented in Figure 3C(I)–C(III). In this figure, G_Fis_-GV presented a smaller contact angle, assuming a greater affinity and thus a greater magnitude of current. This also explains the behavior of the other devices, where G_Sig_-GV presented the second smaller contact angle, closer to the G_Syn_-GV contact angle. For these reasons, GSig-GV and GSyn-GV presented smaller magnitude of current, close to each other, but slightly bigger for G_Sig_-GV. Additionally, the proposed redox reaction is represented in Figure 6B, which includes the loss of one H^+^ and one electron from each phenol group in the oxidation process and the opposite in the reduction process.

Furthermore, SWV was used with CA in the same concentration as used in the previous analyses, as presented in Figure S2. This experiment was carried out utilizing a step (*s*) of −5.0 mV, an amplitude (*a*) of 20 mV, and a frequency (*f*) of 25 Hz, in a cathodic scan. In Figure S2, the peak potential of the sensors had close values: −58.2, −68.3, and −63.2 mV for G_Fis_-GV, G_Sig_-GV, and G_Syn_-GV, respectively. However, the current magnitude of G_Fis_-GV is higher than that of the others, but its error is larger, with values of −4.73 (±0.2029) μA, followed by G_Sig_-GV with −2.93 (±0.05545) μA and G_Syn_-GV with −2.85 (±0.09773) μA. Because of these values of current magnitude and for their smaller deviation, G_Fis_-GV and G_Sig_-GV were selected to continue this work.

The pH value of the electrolyte solution was also optimized for each device, as well as the technique parameters (*a*, *s* and *f*), and the optimal values are shown in Table S1. All of those parameters were close, except for *a*, as also demonstrated in that table.

**Figure 7:**
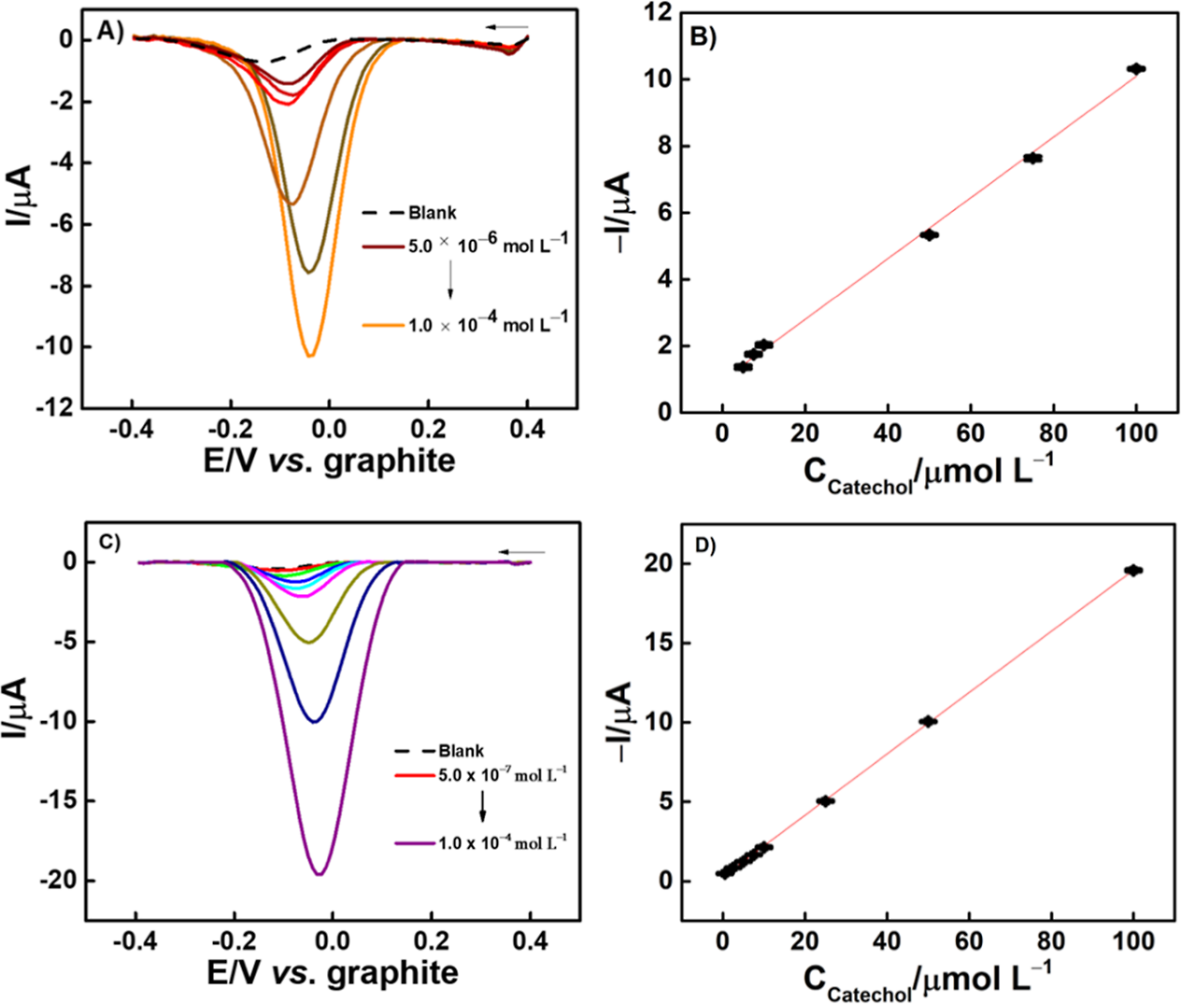
Square wave voltammograms obtained by (A) G_Fis_-GV, in the presence of varying concentrations of CA: 5.00, 7.50, 10.0, 50.0, 75.0, and 100 μmol L^–1^, in 0.2 mol L^–1^ PB (pH 8.0); *s* = −0.010 V, *a* = 0.030 V and *f* = 35 Hz. (B) Analytical curve of G_Fis_-GV; (C) G_Sig_-GV, in the presence of varying concentrations of CA: of 0.500, 2.50, 5.00, 7.50, 10.0, 25.0, 50.0, and 100 μmol L^–1^, in 0.2 mol L^–1^ PB (pH 8.0); *s* = −0.009 V, *a* = 0.060 V, and *f* = 35 Hz. (D) Analytical curve of G_Sig_-GV.

Next, the variation of response with the increase in CA concentration was investigated by SWV, using the optimal values mentioned (Figure 7A,B for G_Fis_-GV and G_Sig_-GV, respectively). Analytical curves were obtained with the data collected, as shown in Figure 7B,D. Both devices responded linearly, with G_Fis_-GV having a smaller linear range: from 5.00 to 100 μmol L^–1^, while G_Sig_-GV ranged from 0.500 to 100 μmol L^–1^ of CA. Besides, the G_Sig_-GV sensor was the only one that demonstrated to be robust enough to sustain all analyses in a unique device, as presented in Figure 7C, while the experiment with G_Fis_-GV needed to be done using different sensors, with three sensors per concentration. G_Sig_-GV presented a greater linearity and lower limit of detection (LOD) than G_Fis_-GV, which was calculated by 3.0 × SD blank/slope, using a total of 10 blanks. The representative equation, for G_Sig_-GV, is *I*_p_ = 0.193C_CA_ (±9.620 × 10^–4^) + 2.88 × 10^–7^ (±3.924), with a *r*^2^ of 0.999 and LOD of 1.2 × 10^–7^ mol L^–1^. For G_Fis_-GV, the equation is *I*_p_ = 0.0912C_CA_ (±2.180 × 10^–3^) + 9.79 × 10^–7^ (±1.202), *r*^2^ of 0.997 and LOD of 1.4 × 10^–6^ mol L^–1^. The greater sensitivity of G_Sig_-GV is possibly specified by the presence of the smaller clusters in its surface, demonstrated in Figure 2B(2),B(3) in SEM analysis. This magnitude differences between the clusters in G_Sig_-GV and G_Fis_-GV provided the largest surface area and consequently a greater magnitude of current.

Both voltammograms, Figure 7A,C, showed peak potential shifts. For G_Fis_-GV (Figure 7A), the shift occurs toward more positive values with the concentration increase, possibly due to the presence of pores, as previously demonstrated by SEM and EIS analysis, which can interfere with the availability of active sites and generate peak potential differences in each device utilized for the construction of this analytical curve. This rationale is different for G_Sig_-GV (Figure 7C), where it was possible to use only one sensor for all these analyses, and because of that, an adsorption process may have occurred on its surface, resulting in a variation of the peak potential by increasing concentrations. Also, these devices showed attractive values of RSD for repeatability and reproducibility (*n* = 9), with values of 3.18% for G_Fis_-GV and 1.18% for G_Sig_-GV in repeatability and 12.1% and 8.88% for G_Fis_-GV and G_Sig_-GV in reproducibility, represented in Figure S3A as a bar graphic, including their respective voltammograms in Figure S3B. Furthermore, the devices were applied in real samples for CA detection, utilizing the spike and recovery method with distribution water. The results were satisfactory for both sensors, achieving recovery values from 95.1 to 101% for G_Fis_-GV and from 99.9 to 104% for G_Sig_-GV, as shown in Table S2.

Table 1 shows some devices from the literature that have similarities with the proposal ePAD, such as the presence of graphite in their composition or even the use of a paper substrate for CA detection in specific type samples, such as water. Almost all the examples, with the exception of the work of the Yang et al.,^45^ used distribution water or tap water as samples. Analyzing these data, it is possible to assert that the properties arising from these evaluated materials are adequate for the promising performance of sensors, when compared to those in the literature. Among the mentioned devices, those that include paper as a substrate are PD-CFB^46^ and the paper electrode of Pradela-Filho et al.^35^ In both cases, the values of the LOD are higher than those calculated in this work. In comparison to sensors that did not use paper as a substrate, G_Sig_-GV achieved a lower value of LOD, except for AuNPs/EGPE,^47^ which had an even smaller value of LOD. This device and G_Sig_-GV obtained a linear range starting at smaller concentrations than the other examples shown. However, NFG-BAC/GCE^45^ and PD-CFB^46^ presented a wider linear range compared to the one achieved in this work. Regarding sensitivity, G_Sig_-GV was the most interesting, followed by NFG-BAC/GCE^45^ and PD-CFB.^46^ In this context, G_Fis_-GV showed a smaller sensitivity but the paper electrode presented smaller values. Thus, the proposed sensors demonstrated comparable performance to those in the literature. It is also interesting to highlight that the production of G_Fis_-GV and G_Sig_-GV has low added value, mainly when compared to alternatives, and is easy to produce.

**Table 1:** Comparison of G_Sig_-VV and G_Fis_-VV with Devices Found in the Literature for CA Detection^a^

sensor	technique	linear range (μmol–L^–1^)	sensitivity (μA L/μmol)	LOD (μmol L^–1^)	real samples	ref
PD-CFB	LSV	50 to 1100	0.129	10	distribution water	(46)
NFG-BAC/GCE	DPV	1.0 to 1000	0.184	0.20	lake water	(45)
paper electrode	LSV	10 to 1000	0.0200	9.0	tap water, vaginal cream and tablet	(35)
AuNPs/EGPE	DPV	0.50 to 100.0	5.36	0.027	river and domestic wastewater	(47)
G_Fis_-GV	SWV	5.0 to 100.0	0.0912	1.4	tap water	this work
G_Sig_-GV	SWV	0.50 to 100.0	0.193	0.12	tap water	this work

a PD-CFB: electrode drawn by pencil on corrugated fiberboard substrate; NFG-BAC/GCE: glassy carbon electrode modified with graphite nanoflake and bamboo activated carbon; paper electrode: graphite ink electrode on filter paper substrate; AuNPs/EGPE: exfoliated graphite paper modified by electrodeposition of gold nanoparticles.

## 4 Conclusions

The morphological characterizations revealed some structural differences between the graphite brands analyzed. In the SEM images, we observed variations in size, forms, and agglomeration for each ink composition, with slightly smaller clusters in G_Sig_-GV. The obtained angles were enough to categorize all devices as hydrophilic, but with a slight variation of angle variation, suggesting different surface structures for each. The devices were also electrochemically characterized: the calculated electroactive areas were slightly different, as were the electron transfer resistance seen in EIS analyses. Besides, for CV and SWV techniques, G_Fis_-GV and G_Sig_-GV achieved higher current magnitudes in CA detection, and the work continued with only these two ePAD. Furthermore, G_Sig_-GV presented a wide linear range in the analytical curve: from 0.500 to 100 μmol L^–1^, while G_Fis_-GV ranged from 5.00 to 100 μmol L^–1^ of CA. The sensitivity of G_Sig_-GV was also higher, with a value of 0.193 and a smaller LOD of 1.2 × 10^–7^ mol L^–1^, compared to G_Fis_-GV results of 0.0912 and 1.39 × 10^–6^ mol L^–1^, respectively. Therefore, even though both devices produced satisfactory results for CA detection in real samples, the Sigma-Aldrich graphite, under these conditions of lab-made ink preparation, presented a more efficient, stable, and sensitive device for CA detection.

## Supporting Information Available

Images of the respective sensors: G_Fis_-GV, G_Sig_-GV, and G_Syn_-GV, Square wave voltammograms, comparison of RSD values of repeatability and reproducibility, comparison of the studied pH values and technique parameters, and spike and recovery data of the distribution water sample for G_Fis_-GV and GSig-GV (PDF)
