# Supplementary material for: Properties Evaluation of Different Graphite Brands through the Development of Electrochemical Sensors for Phenolic Compounds Detection
Source: ACS Omega. 2025 Feb 4;10(6):6047–55. doi: 10.1021/acsomega.4c10281 (PMC11840762; doi:10.1021/acsomega.4c10281)
Supplement: Supplementary file 1 — ao4c10281_si_001.pdf [file ao4c10281_si_001.pdf]

Properties evaluation of different graphite brands through the development of electrochemical sensors for phenolic compounds detection

Amanda Neumann[a], Luiz Otávio Orzari[a,b], Juliano Alves Bonacin[c] and Bruno Campos Janegitz[a,b,*]

[a] *Laboratory of Sensors, Nanomedicine, and Nanostructured Materials, Federal University of São Carlos, 13600-970, Araras, SP, Brazil*

[b] *Department of Physics, Chemistry and Mathematics, Federal University of São Carlos, 18052-780, Sorocaba, SP, Brazil*

[c] *Institute of Chemistry, University of Campinas, 13083-859, Campinas, Sao Paulo, Brazil*

*Corresponding author: *brunocj@ufscar.br*

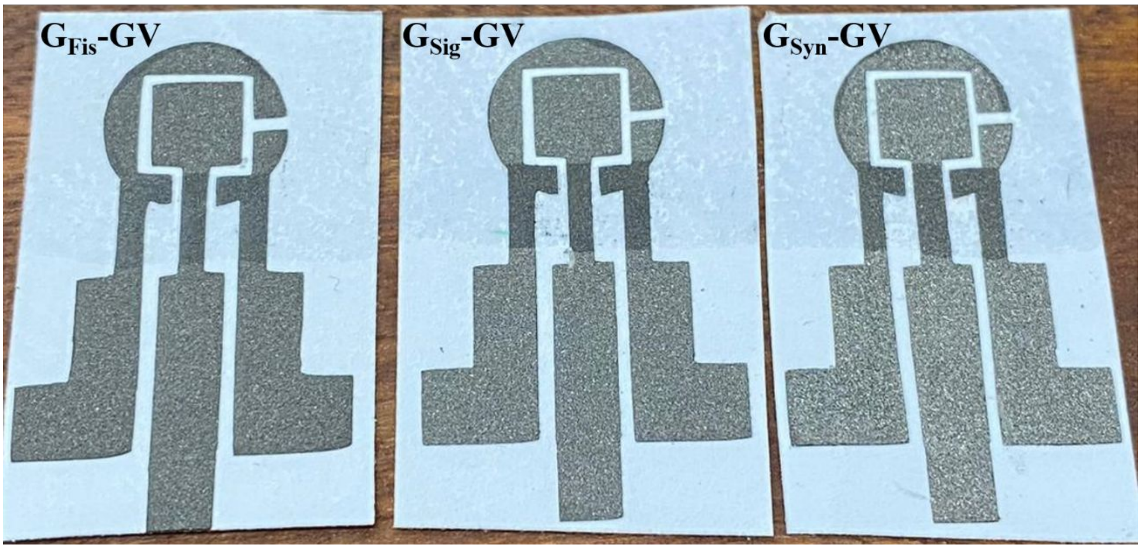

Fig. S1. Images of the respective sensors of G_{Fis}-GV, G_{Sig}-GV and G_{Syn}-GV

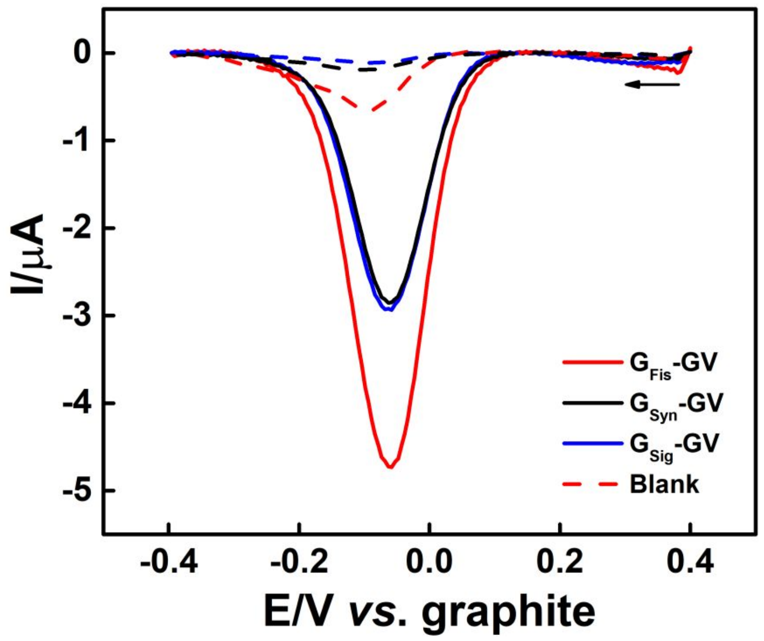

Fig. S2. Square wave voltammograms obtained by G_{Fis}-VV (red), G_{Sig}-VV (blue) and G_{Syn}-VV (black), in the absence and presence of 1.0×10^{-4} mol L^{-1} CA, in 0.2 mol L^{-1} PB (pH 7.0); $s = -0.0050$ V, $a = 0.020$ V and $f = 25$ Hz

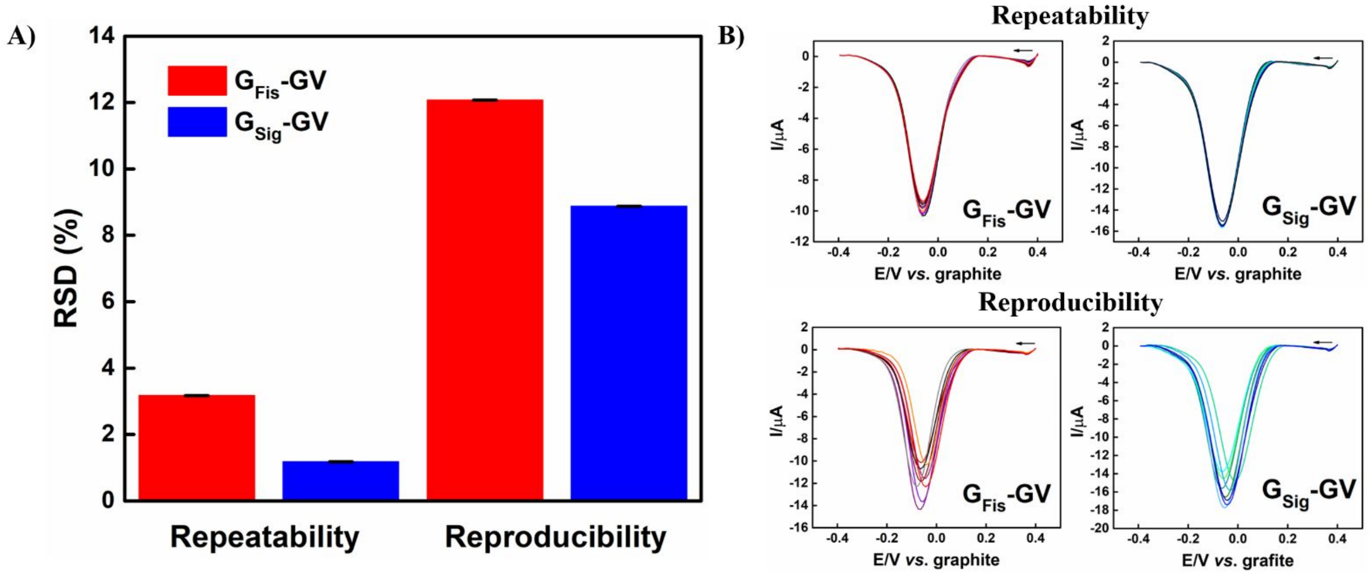

Fig. S3. (A) Comparative of RSD values of repeatability and reproducibility for G_{Fis}-GV (red) and G_{Sig}-GV (blue), (n = 9); (B) Square Wave Voltammograms obtained by G_{Fis}-GV, in the presence of 1.0 × 10^{-4} mol L^{-1} CA, in 0.2 mol L^{-1} PB (pH 8.0); *s* = –0.010 V, *a* = 0.030 V and *f* = 35 Hz, and by G_{Sig}-GV, in the presence of 1.0 × 10^{-4} mol L^{-1} CA, in 0.2 mol L^{-1} PB (pH 8.0); *s* = –0.009 V, *a* = 0.060 V and *f* = 35 Hz

Table S1. Comparison of the studied pH values and technique parameters

**Sensor**	**pH**	**Step (V)**	**Frequency (Hz)**	**Amplitude (V)**
G_{Sig}-VV	8.0	–0.0090	35	0.060
G_{Fis}-VV	8.0	–0.010	35	0.030

Table S2. Spike and recovery data of the distribution water sample for G_{Fis}-GV and G_{Sig}-GV

**Device**	**Sample**	**Addition ($\mu mol\ L^{-1}$)**	**Recovery ($\mu mol\ L^{-1}$)**	**Percentage (%)**
$G_{Fis\text{-}GV}$	A	25.0	25.3 ± 0.084	101
	B	50.0	47.6 ± 0.012	95.1
	C	75.0	75.5 ± 0.069	101
$G_{Sig\text{-}GV}$	A	25.0	25.9 ± 0.052	104
	B	50.0	50.0 ± 0.11	99.9
	C	100	100 ± 0.20	100
